# Trends in male semen parameters (2011–2018): a large-scale retrospective analysis of 5,886 cases based on the fifth edition WHO manual

**DOI:** 10.3389/fpubh.2026.1777051

**Published:** 2026-02-25

**Authors:** Longlong Fu, Fang Fang, Fang Zhou, Ying Guo, Shusong Wang, Jing Ma, Yiqun Gu, Wenhong Lu, Ying Liu

**Affiliations:** 1 Reproductive Health Research Centre/Human Sperm Bank, NHC Key Laboratory of Frontiers and Technologies in Reproductive Health, National Research Institute for Family Planning, Beijing, China; 2 Institute of Pediatric Research, Children’s Hospital of Soochow University, Suzhou, Jiangsu, China; 3 Hebei Key Laboratory of Reproductive Medicine, Hebei Reproductive Health Hospital, Shijiazhuang, China

**Keywords:** air pollution, environmental pollutants, male fertility, retrospective cohort analysis, semen parameters

## Abstract

**Importance:**

Global reports suggest declining sperm quality, but data from Asian populations under standardized conditions are limited. Investigating trends in China is critical for understanding modifiable factors affecting male fertility.

**Objective:**

To assess decade-long trends in semen quality among healthy Chinese men and evaluate associations with environmental factors. Design, Setting, and Participants: Retrospective cohort analysis of 5,886 semen samples from healthy sperm donors (aged 20–45 years) recruited between 2011 and 2018 at the Beijing Human Sperm Bank. All procedures adhered strictly to WHO 5th Edition laboratory standards. Main Outcomes and Measures: Annual trends in semen volume, sperm concentration (SC), total sperm count (TSC), progressive motility (PR), total motility (PR + NP), and percentage of progressive motility (PPR). Associations between semen parameters and environmental pollutants (SO₂, NO₂, PM10, PM2.5, waterborne PI/AN) were evaluated using Spearman correlation.

**Results:**

From 2011 to 2018, significant improvements occurred across key parameters: SC increased by 12.3% (78–96.5 × 10^6^/mL; *p* < 0.05 in 2013, 2018); TSC increased by 18.7% (200–283.5 × 10^6^/ejaculate; *p* < 0.05 from 2014 to 2017); PR and PPR also significantly improved (*p* < 0.05 in multiple years). Negative correlations were observed between pollutants and semen quality: SO₂, NO₂, and PM10 inversely correlated with TSC (*ρ* = −0.719 to −0.929; *p* ≤ 0.045) and PPR (*ρ* = −0.826 to −0.922; *p* ≤ 0.011). Water pollutants (PI, AN) similarly correlated with reduced semen volume, TSC, and motility (*ρ* = −0.735 to −0.878; *p* ≤ 0.038).

**Conclusions and relevance:**

Contrary to global declines, semen quality significantly improved among healthy Beijing donors from 2011 to 2018. This improvement coincided with aggressive environmental policies (e.g., China’s 2013–2017 Air Pollution Action Plan), suggesting pollution-related sperm damage may be reversible with targeted interventions.

## Introduction

Infertility has emerged as a critical global health challenge, with approximately 17.5% of the adult population suffers from infertility affecting about one in six couples worldwide. Recent epidemiological surveys indicate that male factors contribute to 40–50% of infertility cases (1). Over the past four decades, accumulating evidence from industrialized nations has suggested a concerning decline in human semen quality, particularly characterized by reduced sperm concentration and total sperm count (2). However, significant controversies persist regarding the generalizability of these trends, particularly in Asian populations and under standardized laboratory conditions.

Differences in study populations—ranging from fertile volunteers to military recruits and sperm bank donors—as well as heterogeneity in semen collection and analytical methods may introduce bias. Since its first edition in 1980, the World Health Organization (WHO) Laboratory Manual for the Examination and Processing of Human Semen has undergone multiple revisions (3), each refining protocols for sample collection, analysis of sperm concentration, motility, morphology, and quality-control procedures. Over the years, our team has been working on the promotion and dissemination of the WHO manual in China, and has been carrying out the construction and quality control of Chinese male laboratories (4, 5). The interpretation of longitudinal semen parameter changes remains complicated by methodological inconsistencies across studies. Notably, the transition from WHO 4th to 5th edition introduced critical modifications including updated reference limits, standardized quality control measures, and revised classification of teratozoospermia (5, 6). These methodological shifts may introduce systematic biases when comparing historical data, potentially confounding true biological trends with technical artifacts. In order to provide more valid data on changes in male fertility, we used a single-center retrospective survey of possible changes in semen quality, using the same criteria and based on a large fixed population.

China presents a unique epidemiological landscape for investigating male fertility trends. Rapid urbanization, environmental exposures, and lifestyle transitions have created distinct pressures on reproductive health (7). Nevertheless, existing Chinese studies exhibit marked heterogeneity in cohort selection criteria and laboratory methodologies (8, 9). Notably, the study period coincided with the implementation of large-scale national environmental interventions, particularly the Air Pollution Prevention and Control Action Plan launched in 2013, providing a unique policy-relevant context to explore population-level reproductive health trends. And to avoid the effect of COVID-19 that outbroke in 2019, we analyzed the semen data, in our lab from 2011 to 2018 to discover the trend of sperm quality. By leveraging a large, well-characterized cohort and standardized laboratory procedures, we aim to provide robust evidence on modern semen quality in a major urban Chinese population, clarify environmental determinants of male reproductive health, and inform future surveillance and intervention strategies. Our findings aim to resolve current controversies regarding semen quality trends while providing novel insights into environmental determinants of male reproductive health.

## Materials and methods

### Ethics statement and sample collection

This study was conducted with the approval of Human Subjects Ethics Committee of National Research Institute for Family Planning (NRIFP2023024), and all the study participants provided written consent, agreeing to deliver their own anonymous information for future studies.

All sperm samples were obtained from the Human Sperm Bank of National Research Institute for Family Planning, Beijing China.

### Inclusion criteria

This study was conducted at the Human Sperm Bank, Beijing China, affiliated with the National Research Institute for Family Planning. All participants were healthy, volunteer sperm donors aged 20–45 years, recruited between January 2011 and December 2018. Each candidate completed a physical examination and questionnaire to exclude patients with any underlying genetic or other conditions that clearly affect male fertility (e.g., history of reproductive system diseases, sexually transmitted diseases, reproductive tract infections, cardiovascular diseases, obesity, exposure to gonadotoxic therapies, etc.). In addition, smokers, drug addicts and alcoholics were excluded. All participants should have lived in Beijing for at least 6 months.

### Methods of semen collection and semen analysis

Candidates are asked to collect the semen sample after 2–7 days of abstinence. The semen sample is collected by masturbation in a sterile container prepared by the sperm bank.

Semen samples were collected by masturbation in a designated private room within the sperm bank facility. Samples were delivered to the laboratory within 1 h of collection and maintained at 37 °C during liquefaction. All semen analyses were conducted in strict accordance with the World Health Organization (WHO) Laboratory Manual for the Examination and Processing of Human Semen, 5th Edition (2010). Parameters assessed included semen volume, sperm concentration, total sperm count, progressive motility, and total motility. Volume: Measured using a pre-weighed sterile graduated pipette (±0.1 mL accuracy). Sperm Concentration: Determined via Neubauer hemocytometer after 1:20 dilution with saline. Two independent counts per sample were averaged; discrepancies >10% triggered reanalysis. Motility: Assessed manually under phase-contrast microscopy (400 × magnification). A minimum of 200 spermatozoa were classified as progressive (PR), non-progressive (NP), or immotile (IM). All assessments were performed using Makler counting chambers and phase-contrast microscopy by trained andrologists who underwent annual re-certification.

### Semen cryopreservation and thawing protocol

The semen cryopreservation procedure strictly adhered to the standard operational protocols established in the sperm bank (10). The cryoprotectant utilized was GEYC, with the following composition per 100 mL: 1.5 g glucose, 1.3 g sodium citrate, 1.3 g glycine, 15 mL glycerol, and 20 mL fresh egg yolk.

Semen samples were mixed with GEYC at a 2:1 ratio (semen: GEYC) and incubated at 30–35 °C for 5 min. Subsequently, slow freezing was initiated using a programmable freezer. The cooling protocol comprised the following stages: Cooling from 20 °C to −6 °C at a rate of 1.5 °C/min; rapid cooling from −6 °C to −100 °C at a rate of 6 °C/min; holding at −100 °C for 30 min. Following this protocol, the samples (in tubes) were immediately transferred to liquid nitrogen for storage. After a minimum storage period of 24 h in liquid nitrogen, the cryopreserved samples were thawed. Thawing involved incubation at 37 °C for 5 min, after which sperm quality assessment was performed.

The cryopreservation recovery percentage of progressive motility sperm (PPR) serves as the most reliable indicator for assessing sperm cryotolerance.

### Quality control and quality assurance

To ensure the accuracy and consistency of semen analysis, a comprehensive quality control (QC) and quality assurance (QA) system was implemented, consisting of:

#### Internal quality control (IQC)

Daily calibration of pipettes, microscopes, and counting chambers was performed. Analysts routinely measured control samples with known sperm concentration and motility to monitor intra- and inter-technician variability. The coefficient of variation (CV) for key parameters (e.g., sperm concentration, motility) was maintained below 15%.

#### External quality assessment (EQA)

The laboratory participated in the External Quality Control Programme for Semen Analysis administered by the National Research Institute for Family Planning & WHO Collaborating Center for Human Reproductive Health Research (CHN-063). Blind duplicate samples were assessed biannually, and results were compared with national and international reference laboratories.

### Environmental exposure assessment

To assess the influence of environmental factors on semen quality, we utilized the “Beijing Environmental Status Bulletin” published by the Beijing Municipal Bureau of Ecology and Environment for all years^1^ for environmental data. This official network provides real-time and historical air quality data across the city and is recognized as the authoritative source for environmental surveillance in Beijing.

### Statistical analysis

Statistical analyses were performed using SPSS 20.0.0. Continuous variables were compared using Mann–Whitney *U* test. In correlation analysis, the median is selected as the cut-off value and Spearman correlation analysis is used. Statistical significance was considered at *p* values <0.05, denoted as (*p < 0.05, **p < 0.01, and ***p < 0.001).

## Results

### Between 2011 and 2018, sperm quality showed significant improvement

**Figure 1:**
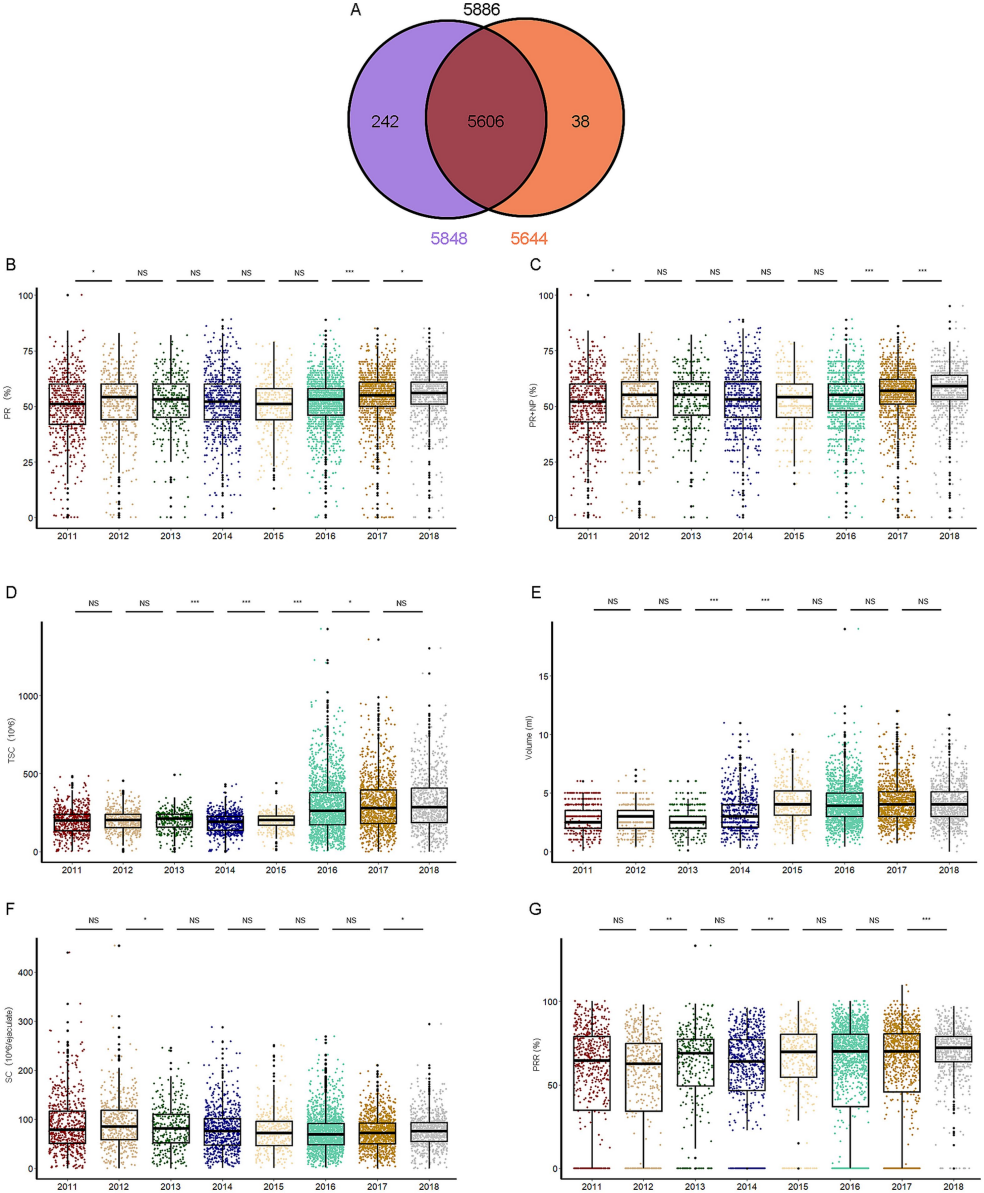
The semen parameters of the samples. **(A)** Flowchart showing sample sizes for different parameters: 5,848 samples for progressive motility (PR), total motility (PR+NP), total sperm count (TSC), volume, and sperm concentration (SC); 5,644 samples for percentage of progressive motility (PPR). (B-G) Annual variations in sperm parameters (2010–2018): **(B)** PR, **(C)** total motility, **(D)** TSC, **(E)** volume, **(F)** SC, and **(G)** PPR. Data are shown as mean ± SEM. *p < 0.05, **p < 0.01; ***p < 0.001; NS, not significant.

We recruited 5,886 sperm samples from Beijing, China, and evaluated key parameters: progressive motility (PR), total motility (PR + NP), total sperm count (TSC), volume, sperm concentration (SC), and percentage of progressive motility (PPR). Of these, 5,848 samples were used for analyzing the first five parameters, and 5,644 samples for the latter parameter (Figure 1A).

PR and total motility (PR + NP) increased significantly in 2012, 2017, and 2018 (Figures 1B,C). TSC showed significant growth from 2014 to 2017 (Figure 1D), while volume increased notably in 2014–2015 (Figure 1E). SC rose significantly in 2013 and 2018 (Figure 1F), and PPR increased in 2013, 2015, and 2018 (Figure 1G).

### Association between sperm parameters and environmental factors

Given the overall improvement in sperm parameters, we investigated their potential association with environmental factors, using the median values of sperm parameters as the reference (Table 1). The environmental factors included: (1) air pollutants: sulfur dioxide (SO_2_) and nitrogen dioxide (NO_2_) emissions, SO_2_, NO_2_, particulate matter 10 (PM10), annual average pH of atmospheric precipitation (pH), and acid rain frequency (ARF); (2) water quality indicators: ammonia nitrogen (AN) emissions, Permanganate index (PI), ammonia nitrogen (AN) in surface water, and chemical oxygen demand (COD) emissions from wastewater (Table 1).

**Table 1:** The median values of sperm parameters and the environmental factors.

Year	Volume (mL)	TSC (10^6^/ejaculate)	SC (10^6^/mL)	PR (%)	PR + NP	PRR (%)	PM2.5 (μg/m^3^)	SO₂ (mg/m^3^)	NO₂ (mg/m^3^)	PM10 (mg/m^3^)	pH	ARF (%)	PI (mg/L)	AN (mg/L)	COD (*10^7^ kg)
2011	2.5	200.0	78	51	52	64.4		0.028	0.055	0.114	5.52	9.8	8.55	6.87	19.32
2012	3.0	202.0	85	54	55	62.5		0.028	0.052	0.109	5.34	28.1	7.75	5.91	18.65
2013	2.5	210.0	81	53	55	68.7	0.0895	0.0265	0.056	0.1081	5.38	16	7.89	6.17	17.85
2014	3.0	191.0	75	52	53	64.0	0.0859	0.0218	0.0567	0.1158	5.76	19	8.05	5.94	16.88
2015	4.0	201.0	72	51	54	69.7	0.0806	0.0135	0.05	0.1015	6.33	4.8	7.71	5.68	16.15
2016	3.9	260.7	69	53	55	70.0	0.073	0.01	0.048	0.092	6.43	4.3	7.37	5.4	14.91
2017	4.0	278.7	72	55	57	70.0	0.058	0.008	0.046	0.084	6.75	0	5.97	2.62	11.59
2018	3.4	283.5	96	63	65	72.0	0.051	0.006	0.042	0.078	6.90	0	4.91	0.98	#

#COD data for 2018 were not publicly reported in the official Beijing Environmental Status Bulletin and were therefore unavailable (https://sthjj.beijing.gov.cn/bjhrb/index/xxgk69/sthjlyzwg/1718880/1718881/1718882/index.html).

**Figure 2:**
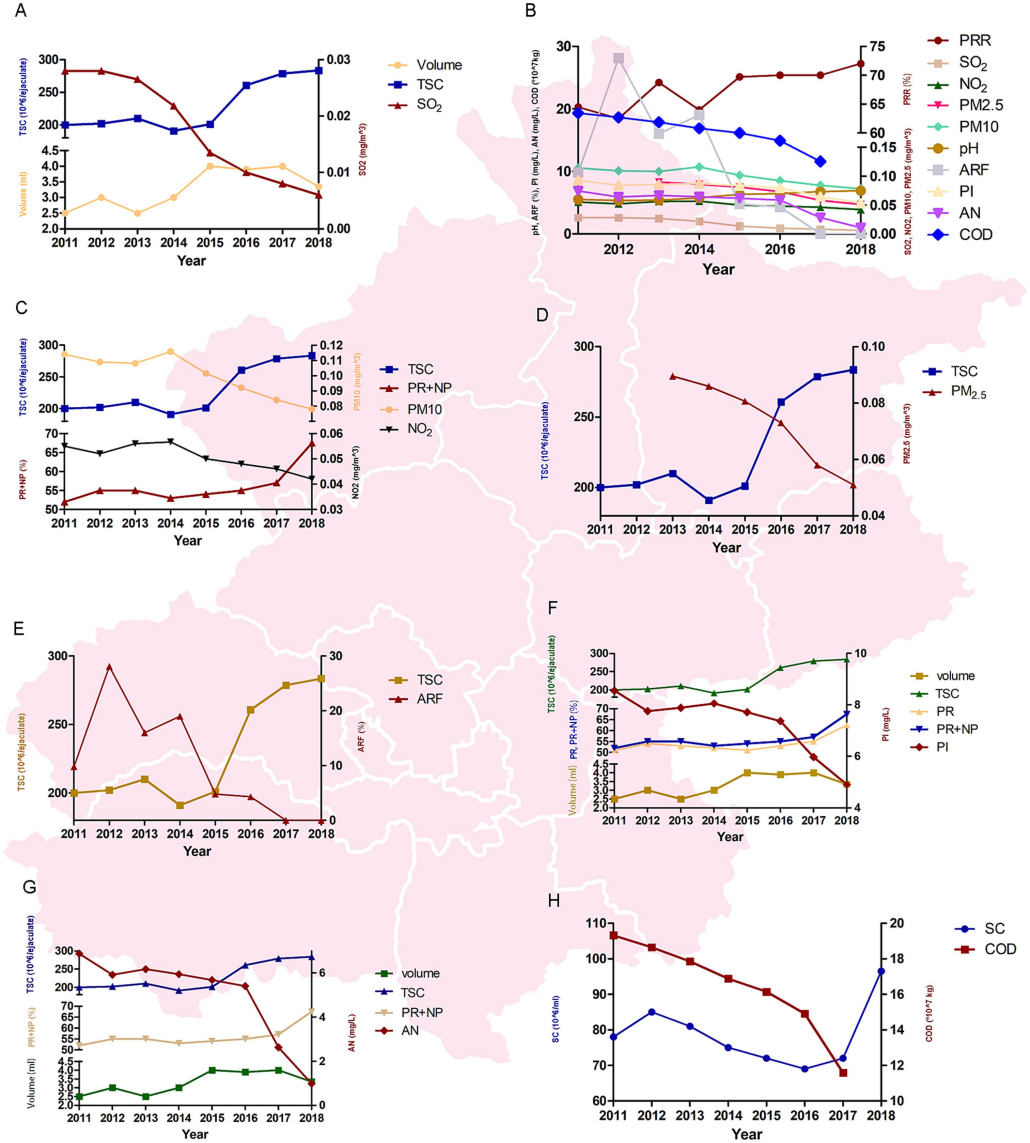
The sperm parameters and environmental factors. **(A)** SO₂ negatively associated with semen volume, total sperm count (TSC), and percentage of progressive motility (PPR). **(B)** Summary of associations for panels C–H. **(C)** NO₂ and PM₁₀ negatively associated with TSC, total motility (PR+NP), and PPR. **(D)** PM₂.₅ negatively associated with TSC and PPR. **(E)** Acid rain frequency (ARF) negatively correlated with TSC and PPR. **(F)** Waterborne lead (PI) negatively correlated with semen volume, TSC, progressive motility (PR), total motility, and PPR. (G) Waterborne ammonia nitrogen (AN) negatively correlated with semen volume, TSC, total motility, and PPR. **(H)** Wastewater chemical oxygen demand (COD) negatively correlated with semen volume and PPR, but positively correlated with sperm concentration (SC).

We found that: (1) ambient SO₂ was significantly negatively associated with semen volume, TSC, and PPR (Figures 2A,B); (2) ambient NO₂ and PM10 were both significantly negatively associated with TSC, total motility (PR + NP), and PPR (Figures 2C,B); (3) ambient PM2.5 was significantly negatively associated with TSC and PPR (Figures 2D,B); (4) the pH of atmospheric precipitation showed a positive correlation with PPR (Figure 2B); (5) ARF was significantly negatively correlated with TSC and PPR (Figures 2E,B); (6) waterborne PI was significantly negatively correlated with semen volume, TSC, PR, total motility (PR + NP), and PPR (Figures 2F,B); (7) waterborne AN was significantly negatively correlated with semen volume, TSC, total motility (PR + NP), and PPR (Figures 2G,B); (8) wastewater COD emissions were significantly negatively correlated with semen volume and PPR, but positively correlated with SC (Figures 2H,B, Table 2).

**Table 2:** The association between sperm parameters and environmental factors.

Sperm parameters	Correlation statistics	SO_2_ (mg/m^3^)	NO_2_ (mg/m^3^)	PM10 (mg/m^3^)	PM2.5 (mg/m^3^)	pH	ARF (%)	PI (mg/L)	AN (mg/L)	COD (*10^7^ kg)
Volume (mL)	CC	−0.732	−0.703	−0.655	−0.551	0.703	−0.640	−0.752	−0.800	−0.844
	*p* value	0.039	0.052	0.078	0.257	0.052	0.087	0.032	0.017	0.017
	*N*	8	8	8	6	8	8	8	8	7
TSC (10^6^/ejaculate)	CC	−0.719	−0.833	−0.929	−0.829	0.619	−0.731	−0.881	−0.786	−0.607
	*p* value	0.045	0.010	0.001	0.042	0.102	0.040	0.004	0.021	0.148
	*N*	8	8	8	6	8	8	8	8	7
SC (10^6^/mL)	CC	0.241	0.072	0.072	0.029	−0.287	0.283	0.072	0.120	0.829
	*p* value	0.565	0.866	0.866	0.957	0.490	0.497	0.866	0.778	0.021
	*N*	8	8	8	6	8	8	8	8	7
PR (%)	CC	−0.539	−0.627	−0.663	−0.696	0.410	−0.400	−0.735	−0.699	−0.382
	*p* value	0.168	0.096	0.073	0.125	0.313	0.326	0.038	0.054	0.398
	*N*	8	8	8	6	8	8	8	8	7
PR + NP (%)	CC	−0.687	−0.756	−0.854	−0.754	0.537	−0.577	−0.878	−0.805	−0.593
	*p* value	0.060	0.030	0.007	0.084	0.170	0.134	0.004	0.016	0.161
	*N*	8	8	8	6	8	8	8	8	7
PRR (%)	CC	−0.904	−0.826	−0.922	−0.928	0.898	−0.958	−0.826	−0.778	−0.811
	*p* value	0.002	0.011	0.001	0.008	0.002	0.000	0.011	0.023	0.027
	*N*	8	8	8	6	8	8	8	8	7

CC: correlation coefficient, *N*: number of the years.

## Discussion

This study conducted a large-scale retrospective analysis of 5,886 semen samples from healthy sperm donors in the Beijing area (2011–2018). Revealing significant improvements in key semen parameters: sperm concentration (SC) and total sperm count (TSC) increased by 12.3 and 18.7%, respectively, from 2011 to 2018, contrary to the previously reported global decline trend (2). Levine et al.’s (2) comprehensive review and meta-regression of 244 estimates from 1973 to 2011, found that sperm concentration declined by approximately 0.70 million/mL per year—an overall drop of more than 50%—and total sperm count by 2.23 million per year (about 0.75% annually), trends that were most pronounced among unselected Western men. But the Levine et al.’s (2) meta-regression captured four decades and multiple WHO guideline revisions, weighting studies by standard error and adjusting for numerous covariates, yet remained susceptible to between-center and between-era variability in lab practices. Our findings were rigorously standardized according to the fifth edition of the World Health Organization (WHO) criteria, eliminating potential biases caused by differences in testing methods. This study accurately assessed the annual trends in semen parameters over the past decade in a fixed population, providing a reliable basis for understanding changes in population fertility. Cargnelutti et al. (11) in Rome, conducted a retrospective analysis of 3,329 men—divided between idiopathic infertility patients and healthy controls—whose semen was assessed from 2010 to 2019 using consistent WHO 2010 protocols and personnel. They observed no significant change in total sperm number over that ten-year span, and identified body mass index, smoking, and infertility history (but not calendar year) as the principal drivers of inter-individual variability (11). The study thereby minimizing analytical heterogeneity but perhaps missing longer-term environmental influences. Our donors were young (20–45 years old), healthy, and non-smokers, minimizing common confounding factors such as age and comorbidities often encountered in infertility clinical studies. Several methodological and contextual factors likely account for these divergent outcomes.

Our Beijing cohort, analyzed under a single-center, highly standardized WHO 5th-edition framework, spans a period of intensive air and water pollution control measures—most notably the 2013–2017 “Air Pollution Prevention and Control Action Plan”—which coincides with measurable gains in sperm concentration, count, and motility. This is consistent with the findings of Zhang et al. (9), who observed a temporary improvement in semen quality during the period of reduced air pollution before and after the pollution control period in 2017–2018. However, due to an unexpected rise in ozone (O₃) levels during this period, the improvement was short-lived. Notably, Zhang et al. (9) found a significant inverse relationship between O₃ and sperm concentration. This finding underscores the need to pay particular attention to ozone levels when formulating pollution control policies, as changes in atmospheric conditions may have unintended consequences for reproductive health. In contrast, Liu et al. (12) found that semen quality in Henan Province continued to decline from 2009 to 2019, a trend that may reflect long-term exposure to pollutants without significant mitigation measures. Their study suggests that factors such as environmental pollutants, obesity, and lifestyle changes may be contributing to the decline in semen quality. The decline in sperm concentration reported in their study (from 62 million/mL in 2009 to 32 million/mL in 2019) is comparable to other reports from Shandong and global studies, indicating that deteriorating sperm quality is a broader regional and international trend.

Airborne particulate matter (PM10) and heavy metals may disrupt spermatogenesis homeostasis. Particulate matter (PM10) is rich in cadmium (Cd) and lead (Pb), which accumulate in testicular tissue and generate hydroxyl radicals (•OH) through Fenton-type reactions, leading to double-strand DNA breaks (13). In animal models, cadmium exposure reduces spermatogonia proliferation by over 40%, and similar damage aligns with the negative correlation we observed between PM10 and total sperm count. PM10 also disrupts the blood-testis barrier by upregulating matrix metalloproteinase-9, degrading tight junction proteins such as tight junction protein-5 and claudin-3 (14). Blood-testis barrier dysfunction facilitates toxin penetration, explaining the decline in TSC during high PM exposure. Previous studies have shown that gaseous pollutants such as sulfur dioxide (SO₂) and nitrogen dioxide (NO₂) impair sperm motility by damaging mitochondrial function: SO₂-derived sulfites inhibit cytochrome c oxidase (complex IV), thereby inhibiting ATP production, which is crucial for flagellar movement (15). Our findings also support this: SO₂ levels were negatively correlated with motility (*ρ* = −0.904, *p* = 0.002). Additionally, SO₂ increases lipid peroxidation by oxidatively depleting glutathione (GSH), thereby damaging sperm membranes. NO₂ induces abnormal DNA methylation at imprinting sites through nitrosative stress, disrupting post-meiotic sperm development (16). Elevated ammonia nitrogen (AN) concentrations in water are associated with reduced semen volume, potentially through inhibition of aquaporin-9 expression in epididymal cells, impairing fluid reabsorption (17). Additionally, high chemical oxygen demand (COD) in wastewater indicates the presence of estrogen-like endocrine disruptors (e.g., alkylphenols), which antagonize androgen receptor signaling in Sertoli cells, thereby affecting lactate production—a critical energy source for germ cell maturation (18).

This study has several limitations. First, due to the retrospective ecological design, associations between annual semen parameters and population-level environmental indicators cannot be interpreted as causal relationships at the individual level. Second, the environmental correlation analysis was based on limited annual observational data, which may reduce statistical power and increase sensitivity to outliers; therefore, these results should be interpreted with caution. Third, although the donor cohort underwent rigorous screening for major confounding factors, several individual-level factors—including body mass index, lifestyle characteristics, occupational exposures, abstinence duration, and seasonal effects—could not be adjusted for. Finally, as this study was conducted at a single center in Beijing, its conclusions may have limited generalizability to other populations. Future multicenter prospective studies will provide more robust evidence regarding the relationship between male semen quality and environmental factors.

## Conclusion

While global meta-analyses report alarming declines in sperm counts, our study reveals a 12.3–18.7% increase in key semen parameters among Beijing sperm donors (2011–2018). This paradox is particularly noteworthy given the temporal overlap with China’s aggressive environmental policy interventions.

## Data Availability

The raw data supporting the conclusions of this article will be made available by the authors, without undue reservation.

## Footnotes

1
https://sthjj.beijing.gov.cn/bjhrb/index/xxgk69/sthjlyzwg/1718880/1718881/1718882/index.html
